# Myxofibrosarcoma of the abdominal wall : A case report and literature review

**DOI:** 10.1016/j.ijscr.2022.107275

**Published:** 2022-06-04

**Authors:** Hazem Beji, Mahdi Bouassida, Mohamed Fadhel Chtourou, Slim Zribi, Mohamed Mongi Moghri, Hassen Touinsi

**Affiliations:** Department of general surgery, Hospital Mohamed Taher Maamouri, Nabeul, Tunisia; University Tunis El Manar, Faculty of Medicine of Tunis, Tunisia

**Keywords:** Myxofibrosarcoma, Abdominal wall, Mass, Case report

## Abstract

**Introduction:**

Myxofibrosarcoma (MFS) is a subtype of soft tissue sarcoma characterized by diffuse infiltration patterns. Myxofibrosarcoma arises often in extremities. Its occurrence in the abdominal wall is extremely rare.

Herein, we present here a case of high-grade MFS of the abdominal wall discovered in a 58-year-old woman complaining of an abdominal mass.

**Presentation of case:**

This report illustrates the case of a female who presented a mass in the right lower quadrant of the abdomen. Abdominal computed tomography (CT) scan revealed a well-circumcised, heterogeneous soft tissue mass. We performed a wide margin excision of the mass. Histology concluded in myxofibrosarcoma of the abdominal wall. Adjuvant radiotherapy was performed.

**Clinical discussion:**

We reported successful surgical treatment for myxofibrosarcoma of the abdominal wall. To our knowledge, this is the second report in English literature.

MFS is a subtype of soft tissue sarcoma with a locally infiltrative behavior.

To ensure the best curative treatment, It is important to excise the tumor with wide margins.

Knowing that MFS has a propensity for local recurrence (16 to 57 %), adjuvant radiotherapy has emerged as an efficient treatment for improving local control.

The role of chemotherapy is controversial and has not shown effects on survival.

**Conclusion:**

Myxofibrosarcoma is a connective tissue neoplasm. Its occurrence in the abdominal wall is extremely rare.

Surgical treatment with large negative margins is the cornerstone of the treatment. Adjuvant radiotherapy is essential in preventing local recurrences.

## Highlights

• Myxofibrosarcoma of the abdominal wall is an extremely rare situation.
• Surgical treatment is the cornerstone of the treatment.
• Adjuvant radiotherapy is an option to prevent relapses.

## 1 Introduction and importance

Myxofibrosarcoma (MFS) is a subtype of soft tissue sarcoma characterized by diffuse infiltration patterns. It represents 5 % of soft tissue sarcomas [1]. It affects essentially the elderly and predominantly touches the extremities [2]. Tumours are classified into three grades, with grade I being locally aggressive, and grades II and III having metastatic potential.

MFS presents a significant propensity for local recurrence following resection (50 %) [3], [4], [5]. Their occurrence in the abdominal wall is extremely rare. To our knowledge, It was described only once in the English literature [6].

Herein, we present here a case of high-grade MFS of the abdominal wall discovered in a 58-year-old woman complaining of an abdominal mass.

This work has been reported in line with the SCARE 2020 criteria [7].

## 2 Presentation of a case

A 58-year-old female, with no comorbidities, complained of a fast-growing abdominal mass evolving since one year. She had no other symptoms. She had no family history of cancer.

On examination, the patient had a tender, non-reducible, non-mobile mass in the right lower quadrant of the abdomen.

Laboratory studies revealed hemoglobin of 11.2 g/dl, platelet count of 230,000/μl, and prothrombin time of 97 %. Renal and hepatic functions were normal.

**Fig. 1:**
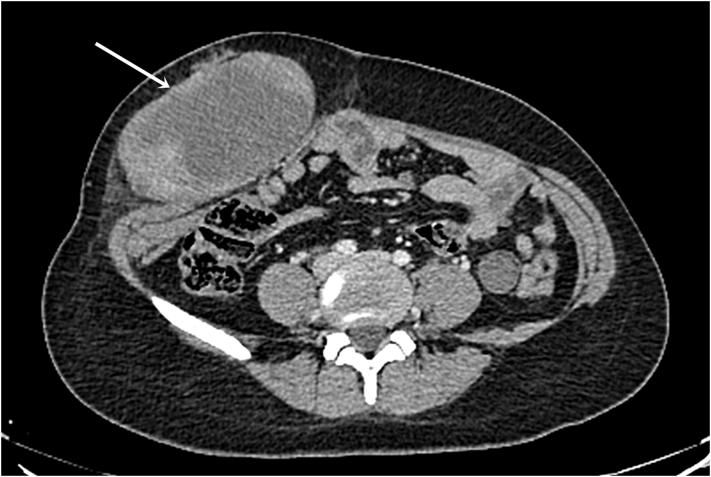
CT scan in the axial plane showing a well-circumcised, heterogeneous soft tissue mass of the abdominal wall (arrow).

**Fig. 2:**
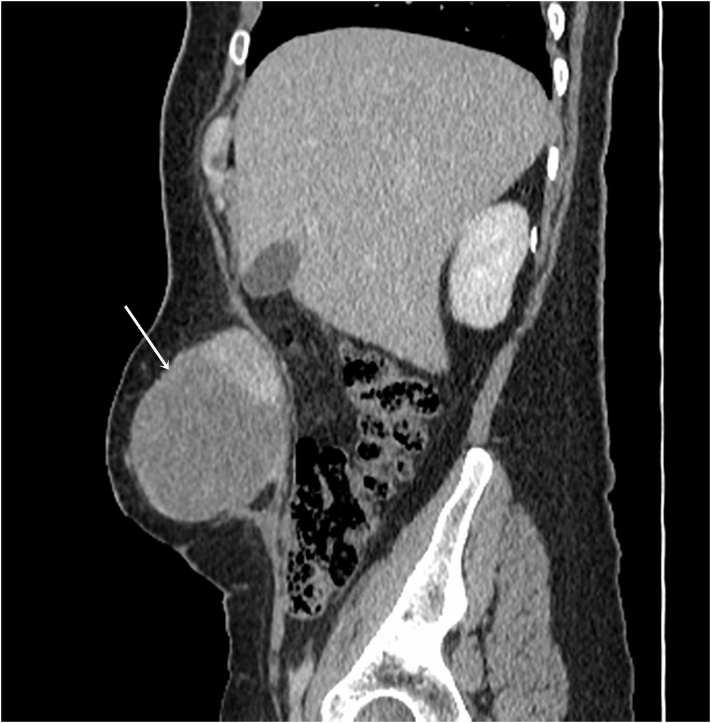
CT scan in the sagittal plane showing the mass of the abdominal wall (arrow).

Abdominal computed tomography (CT) scan revealed a well-circumcised, heterogeneous soft tissue mass measuring 13 × 10 × 8 cm of the abdominal wall (Fig. 1, Fig. 2).

The case was discussed in a multidisciplinary meeting. We considered performing a needle biopsy of the mass or performing surgery directly. We opted for the surgical treatment knowing that the mass was symptomatic and even if there was no malignancy, the mass had to be resected to relieve symptoms.

Surgery was planned and was performed by a 15-year experience surgeon.

We considered the diagnoses of a desmoid tumor and leiomyosarcoma of the abdominal wall.

**Fig. 3:**
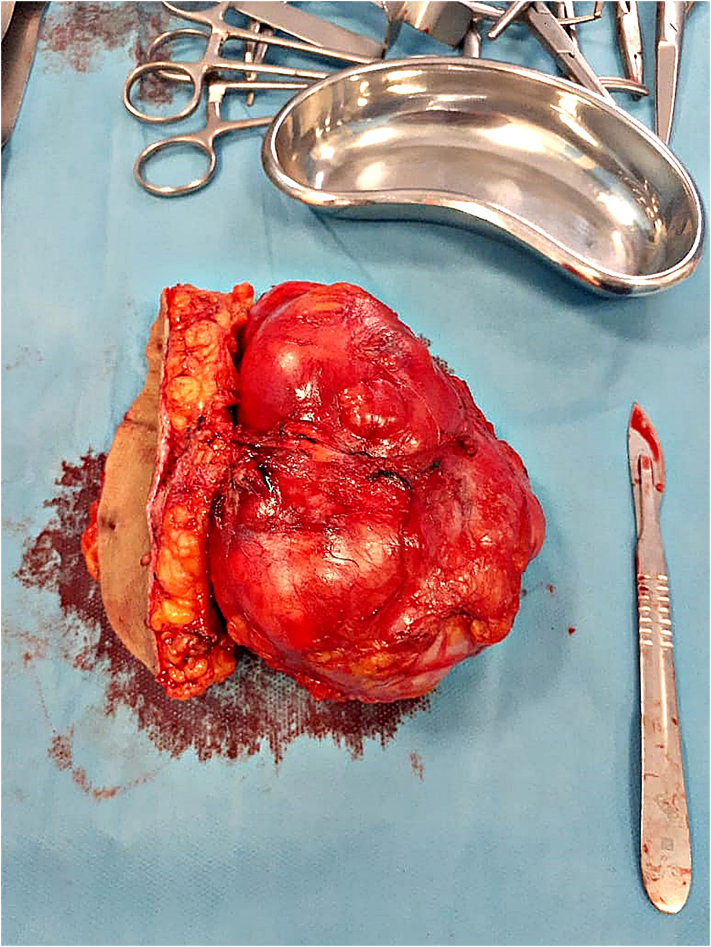
The resection specimen.

We performed an elective incision. We resected the mass and the adjacent skin, dermis, and subcutaneous tissue with a 1 cm margin (Fig. 3). Reconstruction was made by resecting the excessive skin.

**Fig. 4:**
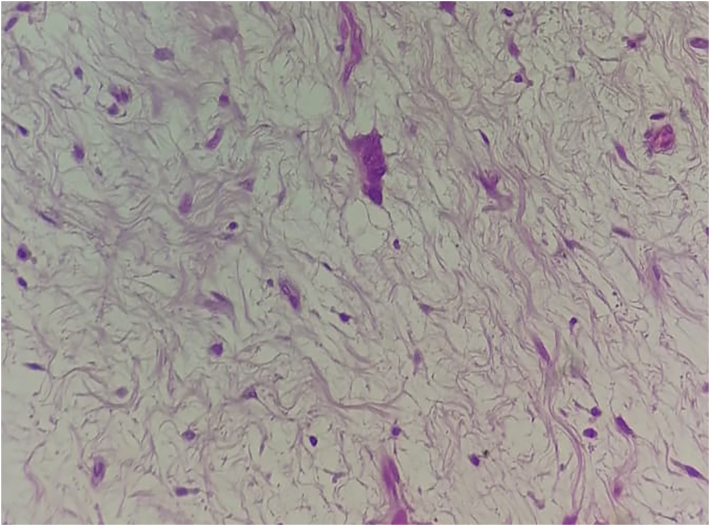
Histopathological slide with hematoxylin and eosin demonstrating high-grade spindle cell tumor with scattered nuclear pleomorphism in a prominent fibromyxoid stroma.

Histopathology showed a high-grade spindle cell tumor with scattered nuclear pleomorphism in a prominent fibromyxoid stroma (Fig. 4). Staining for smooth muscle antibody (SMA), MYOD1, CD34, CD117, and DOG-1 were negative. It confirmed the diagnosis of myxofibrosarcoma grade 3. The resected specimen had negative margins.

The postoperative course was uneventful with a follow-up of two months. Adjuvant radiotherapy was performed to prevent relapses.

## 3 Clinical discussion

We reported successful surgical treatment for myxofibrosarcoma of the abdominal wall. To our knowledge, this is the second report in English literature. Adjuvant radiotherapy was performed to prevent relapses.

The main weaknesses of our work are the short follow-up period of only two months and the non-performance of MRI.

MFS is a subtype of soft tissue sarcoma with a locally infiltrative behavior. It affects generally older patients. Men are affected more frequently than women [1], [8].

The extremities are the most prevalent locations of presentation of MFS (77 %). They can also be seen on the trunk (12 %), and the head and neck area (3 %). MFS is rare in the abdomen, retroperitoneum, skin, breast, and chest wall [1], [9], [10]. Its presence on the abdominal wall is quite uncommon. It was only mentioned once in the English literature [6].

It usually manifests as a slowly growing, painless mass. On computed tomography (CT), MFS present as a heterogeneous soft tissue mass. MRI is the diagnostic modality of choice. It shows a low to intermediate signal on T1-weighted MRI. On T2-weighted MRI, the solid and myxoid components show high signal intensity, with the myxoid component showing higher signal intensity similar to fluids [1].

Needle biopsy of the tumor is a considerable option [6]. We preferred not to perform it in our case to avoid the dissemination of tumor cells. Moreover, the mass was symptomatic and surgical treatment was mandatory.

We considered differentials such as Leiomyoma, Leiomyosarcoma, extra gastrointestinal stromal tumor (E-GIST), and liposarcoma [1], [11].

Intermediate and high-grade sarcoma present a high risk of recurrence (50 %) and distant metastasis. To ensure the best curative treatment, It is important to excise the tumor with wide margins [12], [13], [14], [15].

To date, there is no consensus on the width of surrounding normal tissue that should be excised [16]. Negative tumor margins are essential. It lowers local recurrence and improves long-term survival [17], [18].

Overall survival for patients having localized MFS varies from 61 to 77 % at 5-year [19]. Knowing that MFS has a propensity for local recurrence (16 to 57 %), adjuvant radiotherapy has emerged as an efficient treatment for improving local control [6].

The role of chemotherapy is controversial and has not shown effects on survival [20].

Preoperative radiotherapy can be considered in presence of tumor invasion of an important adjacent structure permitting down-sizing the tumor before surgery. This permits to avoid leaving positive resection margin.

Further prospective cohort studies with a larger sample size could aid in assessing preoperative and postoperative radiotherapy in preventing local recurrence.

In summary, we reported a case of MFS of the abdominal wall treated with surgical excision and adjuvant radiotherapy.

## 4 Conclusion

Myxofibrosarcoma is a connective tissue neoplasm. Its occurrence in the abdominal wall is extremely rare.

Surgical treatment with large negative margins is the cornerstone of the treatment. Adjuvant radiotherapy is essential in preventing local recurrences.

## Sources of funding

This research did not receive any specific grant from funding agencies in the public, commercial, or not-for-profit sectors.

## Ethical approval

Not required.

## Consent

Written informed consent was obtained from the patient for publication of this case report and accompanying images. A copy of the written consent is available for review by the Editor-in-Chief of this journal on request.

## Guarantor

Hazem Beji.

Mahdi Bouassida.

## Provenance and peer review

Not commissioned, externally peer-reviewed.

## Registration of research studies

N/A.

## CRediT authorship contribution statement

Hazem Beji and Mahdi Bouassida did the conception and design of the work, the data collection, and the data analysis and interpretation.

Mohamed Fadhel Chtourou and Slim Zribi did the critical revision of the article.

Mohamed Mongi Mighri and Hassen Touinsi did the final approval of the version to be published.

## Declaration of competing interest

No conflicts of interest.
